# Energy‐restricted interventions are effective for the remission of newly diagnosed type 2 diabetes: A systematic review of the evidence base

**DOI:** 10.1002/osp4.504

**Published:** 2021-05-15

**Authors:** Elizabeth Jacob, Amanda Avery

**Affiliations:** ^1^ Faculty of Science The University of Nottingham Nottingham UK

**Keywords:** calorie restriction intervention, dietary energy restriction, remission, type 2 diabetes

## Abstract

**Background:**

Type 2 diabetes (T2D) is a chronic, progressive disease. Caloric restriction and subsequent weight loss have been associated with both improvements and, in some cases, remission of T2D.

**Aim:**

To systematically review the safety and effectiveness of calorie‐restricted diets on weight change and the remission of T2D.

**Methods:**

Electronic databases were searched. Intervention trials including a calorie restriction, published between 2010 and 2020, evaluating the remission of T2D (HbA1c <6.5% without diabetes medication) were selected. Risk of bias was assessed.

**Results:**

Eight trials met inclusion criteria including four randomized controlled and four single‐arm trials. Three controlled trials found greater remission in the calorie‐restricted arm (*p* < 0.05). A recent diagnosis of diabetes was associated with higher remission rates (75%–80%) with an inverse association between duration of diabetes and rate of remission (*r* = −0.94). A higher level of remission was observed with greater calorie restriction in non‐new diagnosis studies. Greater weight loss was associated with increasing rates of remission (*r* = 0.83). No reported adverse events led to withdrawal from trials. There was great heterogeneity in study design.

**Conclusion:**

Remission rate of T2D achieved through calorie restriction is high and similar to that reported in the bariatric surgery literature. Remission should be the aim at diagnosis and calorie restriction could be used to achieve this. The target weight loss should be >10% body weight in people with obesity. More research is needed into the optimum level of calorie restriction and the support required for long‐term remission. National guidelines should be updated to reflect recent evidence.

## 1 INTRODUCTION

The prevalence of type 2 diabetes (T2D) has increased over the past 20 years with global prevalence rates (in people aged 20–79 years) reaching 9.3% in 2019.^1^ A more recent concern is that T2D is a risk factor for a worse outcome in those with COVID‐19 infection. The mortality rate for those with diabetes or uncontrolled hyperglycemia was found to be more than four times higher than for those without either of those risk factors.^2^ People with T2D have been found to have significantly higher risk of respiratory distress syndrome and multiple organ injury.^3^, ^4^ The economic costs of T2D are increasing as well as the immeasurable pain and suffering of those with the condition.^5^, ^6^, ^7^, ^8^

The national guidance on the management of T2D mentions a few sentences about dietary advice, with the bulk of the information relating to medications and algorithms to progress through when treating worsening hyperglycemia.^9^ The aim of remission of diabetes is not specifically discussed anywhere in this guideline. Eligibility for bariatric surgery is discussed in the national guidelines for obesity management for those with new‐onset T2D with a body mass index (BMI) greater than 30 or at a lower BMI if people are of Asian origin.^10^ The 7‐year cumulative incidence of achieving partial/complete or prolonged remission in people newly diagnosed with T2D on no diabetic medication with standard care was 1.60%.^11^

A recent review by Hallberg et al.^12^ examined reversal of diabetes by three methods: bariatric surgery, carbohydrate‐restricted diets, and low‐calorie diets. There have been a number of systematic reviews in the field of bariatric surgery^13^, ^14^ and carbohydrate restriction^15^, ^16^ for the treatment of T2D. Kelly and colleagues^17^ conducted a review of studies that examined lifestyle modifications targeting the remission of T2D. However, their definition of remission was broad and included normalization of fasting plasma glucose, HbA1c, an oral glucose tolerance test and normalization of *β* cell function. Taylor^18^ summarized the rationale for the DiRECT study, but the article did not systematically review all the published evidence in the field of energy restriction and diabetes remission.

Lim et al.^19^ demonstrated that insulin resistance and pancreatic *β*‐cell failure could be reversed with an energy‐restricted diet (600 kcal/day). There was an associated decrease in liver and pancreatic triacylglycerol stores. Steven et al.^20^ demonstrated that an 8‐week energy‐restricted diet (624–700 kcal/day) produced a sustainable normalization of fasting plasma glucose to less than 7.0 mmol/L in 40% of the study population at 6 months. Responders were noted to have a shorter duration of diabetes (3.8 ± 1.0 vs. 9.8 ± 1.6 years, *p* = 0.007).

There has been much debate about the definition of reversal and remission of T2DM. Buse et al.^21^ suggested a partial remission to be defined as an HbA1c less than 6.5% and fasting glucose of 5.6–6.9 mmol/L for at least 1‐year duration without pharmacological therapy. Complete remission was defined as having HbA1c in the normal range and fasting glucose less than 5.6 mmol/L for 1 year without pharmacological therapy. A simpler HbA1c criteria based on the American diabetes guidelines of diagnosis of diabetes with remission if HbA1c <5.7% and improvement with HbA1c 5.7%–6.5% with no hypoglycemic medication and a duration of at least 1 year has also been proposed.^22^ Some authors^12^ have removed metformin from the list of medications for glycemic control as this drug has a role in prevention.^23^ For the purposes of this review, as some studies included were of short duration and there is such heterogeneity of definition of remission between studies, a complete or partial remission was defined as an HbA1c less than 6.5%/48 mmol/mol without the use of diabetes medication.

The aim of this review was to assess the safety and effectiveness of energy‐restricted interventions in achieving remission of T2D. Scalable interventions for the remission of T2D are urgently needed and there has been no in‐depth review of energy restriction conducted for this purpose with a uniform definition of remission. The primary outcome measure was levels of HbA1c less than 6.5%/48 mmol/mol with no hypoglycemic medications in energy‐restricted dietary interventions. Secondary outcome measures were weight change, reduction in HbA1c levels, and serious adverse events.

## 2 METHOD

A literature search was conducted using the electronic databases of PubMED and Wiley Online. The following search terms were used: #1: “Diabetes” OR “Diabetes Mellitus” OR “Type 2 Diabetes” OR “Non Insulin Dependent Diabetes” OR “T2DM” or “NIDDM”; #2: “Calorie restricted” OR “Very low calorie diet” OR “Lifestyle intervention” OR “Intensive Lifestyle”; #3: “Remission” or “Reversal”.

An advanced electronic search was performed and filters were applied to include only journal articles, publications in the English language, species to be human only, adults and searches were limited to the last 10 years. The final search was conducted on 30 January 2021. The reference lists of relevant publications were hand searched to find any relevant articles. The titles and abstracts were screened against the inclusion and exclusion criteria listed in Table 1. Full‐text articles of the short‐listed articles were then further screened. Inter‐library loans were undertaken when necessary to locate full text articles. A second reviewer independently assessed papers for final eligibility and any disagreement was discussed and resolved.

**TABLE 1:** Inclusion and exclusion criteria

	Inclusion criteria	Exclusion criteria
Publication date	2010–2020	<2010
Language	English	Non‐English
Study design	Randomized controlled trial or intervention study	Cohort, crossover, case–control trial
Population	Adults ≥18 years of age, diagnosis of type 2 diabetes, any duration of diagnosis, any medication regime	Children, animal studies, pre‐diabetes, type 1 diabetes, MODY, non‐diabetes
Intervention	Energy‐restricted dietary intervention	Drug‐based intervention beyond standard care, bariatric surgery, carbohydrate restriction
Control group	Standard care if control present	Bariatric surgery
Primary outcome	HbA1c <6.5% without diabetes medication	Lipid profiles, blood pressure, body mass index, lean mass, changes in medication, quality of life
Secondary outcome	Weight change (kg) and glycemic control (HbA1c%) with both factors clearly related to dietary changes in a multi‐component intervention.	
Study duration	Any duration	

Data were extracted by a single reviewer. The data included author, year, country of origin, duration of study, number of participants, male/female ratio, number of completers of each arm of trial, baseline weight, baseline HbA1c%/mmol/mol, mean duration since diagnosis (unless stated as median), inclusion criteria, intervention and control details, outcome measures of interest and serious adverse events. HbA1c % and mmol/mol were converted where possible, so both could be presented. Mean weight loss and standard deviation and mean change in HbA1c % were calculated from supplementary information for one study.^24^ The Pearson correlation coefficient (*r*‐value) was calculated for a number of variables in the collated results.

The study was reported according to PRISMA statement^25^ (see Figure S1). All supplementary tables are available by contacting the corresponding author.

The Downs and Black^26^ quality assessment tool was used to assess the risk of bias. This tool was chosen as it can be used to compare randomized and non‐randomized controlled trials.

## 3 RESULTS

**FIGURE 1:**
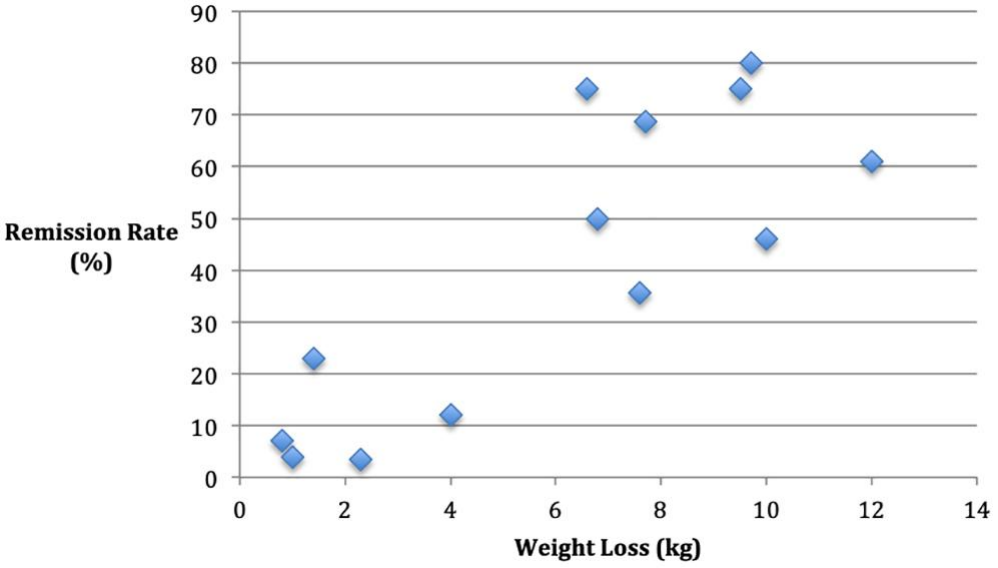
Remission rate compared with weight loss

Preliminary searches found a paucity of randomized controlled trials in this field in the last 10 years, so the decision was made to include single‐arm intervention trials even though they would be at higher risk of bias, to increase the breadth of data to analyze. The literature search generated 660 results. After removal of duplicates, 641 records were screened. Seventeen studies were assessed and finally eight studies were included (see Figure 1). The assessment of bias has been summarized in Table S1, with original scoring in Table S2.

Four publications of randomized controlled trials were found including a 2‐year update for one of the studies. Two of these were retrospective analyses of data. Four single‐arm studies were also included. The total sample size from all of the eight studies was 5764 participants. Energy restriction ranged from prescribed periods of 600 kcal/day to individualized levels between 1200 and 1800 kcal/day for the entire study duration. The length of time since diagnosis ranged from new diagnosis to 5 years. The studies took place in the United Kingdom, India, United States, Denmark, Thailand, and Qatar. Detailed characteristics including interventions and controls (if applicable) for all studies have been described in Table 2.

**TABLE 2:** Summary of main results

Author	*n*	Duration	M/F, completers, mean weight (kg), Baseline HbA1c %/mmol/mol, Mean duration since diagnosis	Inclusion criteria	Intervention/control	Outcome
Lean et al.^,^ ^27^ UK DiRECT trial	298	12 months	Intervention: 83/66, 117/149, 101.0 kg, 7.7/60, 3 years Control: 93/56, 149/149, 98.8 kg, 7.5/58, 3 years	Age 20–65 years, type‐2 diabetes, diagnosis within 6 years, BMI 27–45, no insulin.	Intervention: Withdrawal of antihypertensive and antidiabetic drugs, total diet replacement (825–853 kcal/day for 3–5 months), followed by stepped food reintroduction (2–8 weeks), with structured support for long‐term weight maintenance. Exercise—usual during diet replacement and then increasing steps up to 15,000/day. Control: Best practice care by guidelines.	Difference from baseline and 12 months. Mean weight change (kg): I −10.0, C −1.0 (*p* < 0.0001) Mean HbA1c% change: I −0.9, C +0.1 (*p* < 0.0001) Mean HbA1c mmol/mol change: I −9.6, C +1.4 (*p* < 0.0001) HbA1c <6.5%/48 mmol/mol without diabetic medications: I 46%, C 4% (*p* < 0.0001)
Lean et al.^28^ UK DiRECT trial (2 year update)	298	24 months	Intervention: 83/66, 116/149, 101.0 kg, 7.7/60, 3 years Control: 93/56, 141/149, 98.8 kg, 7.5/58, 3 years	Age 20–65 years, type 2 diabetes, diagnosis within 6 years, BMI 27–45, no insulin.	Intervention: Monthly 30‐min appointment with dietitian or practice nurse.If >2 kg weight gain, 2–4‐week partial meal replacement.If >4 kg weight gain total diet replacement and food reintroduction and offered orlistat. Control: Best practice care by guidelines.	Difference from baseline and 24 months. Mean weight change (kg): I −7.6, C −2.3 (*p* < 0.0001) Mean HbA1c% change: I −0.5, C 0.0 (*p* = 0.006) Mean HbA1c mmol/mol change: I −5.2, C +0.4 (*p* < 0.01) HbA1c <6.5%/48 mmol/mol without diabetic medications: I 35.6%, C 3.4% (*p* < 0.0001)
Bhatt et al.^24^ India	12	12 weeks	Intervention: 8/4, 12/12, 84.3 kg, 9.1/76.0, 3.3 years (median)	Type 2 diabetes.	Intervention: 1000 kcal/day meal replacement. Withdrawal of antidiabetic medication except in 2 patients were reduced to basal insulin and metformin. Antihypertensive medication titrated during course. Moderate intensity aerobic and resistance exercise advised from second week.	Difference from baseline and week 12. Mean weight change (kg): I −6.8 Mean HbA1c% change: I −2.4 HbA1c <6.5%/48mmol/mol without diabetic medications: I 50%
Sarathi et al.^29^ India	32	24 months	Intervention: 73.8 kg, 10.6/92.4, New diagnosis	Newly diagnosed diabetes	Intensive lifestyle therapy: 1500 kcal/day, Brisk walking 1 h/day, HbA1c >9.0% started on metformin 500–2000 mg. Those with comorbid condition started on started on insulin followed by metformin +/− DPP4 inhibitors.	Difference from baseline and year 1. Mean weight change (kg): I −6.8 Mean HbA1c% change: I −4.7 Mean HbA1c mmol/mol change: I −27.9 HbA1c <6.5%/48 mmol/mol without diabetic medications: I 75% Difference from baseline and year 2. Mean weight change (kg): I −7.7 Mean HbA1c% change: I −4.7 Mean HbA1c mmol/mol change: I −27.9 HbA1c <6.5%/48 mmol/mol without diabetic medications: I 68.8%
Gregg et al.^30^Wadden et al.^31^ USA	5145	4 years	ILI: 940/1301, 2241/2570, 100.4 kg, 7.3/56.3, 5 years (median) DSE: 936/1326, 2262/2575, 100.6 kg, 7.4/57.4, 5 years (median)	Type 2 diabetes, age 45–76, BMI ≥25 or ≥ 27 if receiving insulin, HbA1c < 11%	Intensive lifestyle‐based weight loss intervention (ILI): weekly group and individual counselling, then 3 sessions per month second 6 months, then twice monthly years 2–4.Energy intake to 1200–1800 kcal/day.Meal replacements offered.Increase physical activity to 175 min/week. Diabetes support and education (DSE): 3 group sessions per year.	Difference from baseline and year 1. Mean weight change: I −8.6%, C −0.7% (*p* < 0.001) HbA1c <6.5%/48 mmol/mol without diabetic medications: I 11.5%, C 2% (*p* < 0.001) Difference from baseline and year 4. Mean weight change: I −4.7%, −0.8% (*p* < 0.001) HbA1c <6.5%/48 mmol/mol without diabetic medications: I 7.3%, C 2% (*p* < 0.001)
Ried‐Larsen et al.^32^Johansen et al.^33^ Denmark	98	12‐month intervention, 12‐month follow‐up.	U‐TURN: 35/29, 62/64, 95.3 kg, 6.6/49.1, 4.7 years Standard care: 20/14, 31/34, 97.6 kg, 6.7/49.7, 5.6 years	Type 2 diabetes, <10 years, ≤2 glucose lowering medications, BMI 25–40.	U‐TURN: Individualized dietary plan, energy restriction first 4 months and then energy balance 8 months. Supervised aerobic and resistance exercise 30–60 min per day, 5 or 6 days a week. Standard care: Pharmaceutical therapy and lifestyle advice from diabetes nurse every third month.	Difference from baseline and 24 months. Mean weight change (kg): I −1.4, C −0.80 (*p* = 0.17) Mean HbA1 mmol/mol change: I +2.7, C +3.2 (*p* = 0.80) HbA1c <6.5% without diabetic medications: I 23%, C 7% (*p* = 0.08)
Ades et al.^34^ USA	12	6 months	Intervention: 8/4, 10/12, 103 kg, 6.8/51, 93 days	Recently diagnose type 2 diabetes (<1 year), overweight and obese individuals, no diabetes medication, HbA1c 6.5%–8.0%, BMI 27–40.	Intervention: Behavioral weight loss program including 24 weekly group sessions with a dietitian. Calorie deficit 500 kcal per day. Exercise: longer distance walking 5–6 days per week.	Difference between baseline and 6 months (data for completers 10/12). Mean weight change (kg): I −9.7 Mean HbA1c% change: I −0.6 Mean HbA1c mmol/mol change: I −7.0 HbA1c <6.5%/48 mmol/mol without diabetic medications: I 80%
Umphonsathien et al.^35^ Thailand	20	10 weeks VLCD, 4 weeks transition, follow‐up 1 year after discontinuation of VLCD.	Intervention: 1/19, 19/20, 8.0/64, 71.9 kg, 2 years (median)	Type 2 diabetes duration <10 years, age 20–60 years, HbA1c ≥6.5%, BMI 23–30.	Intervention: Run in period 600 kcal/day for 10 days over 2 weeks to assess compliance. If over 90% compliance, then 8 weeks of VLCD. 4 weeks transition period with 800 kcal/day week 9 1000 kcal/day week 10 1200 kcal/day week 11, and 1500 kcal/day week 12.	Difference from baseline and week 14 (2 week run in +12 weeks of trial). Mean weight change (kg): I −9.5 Mean HbA1c% change: I −2.2 Mean HbA1c mmol/mol change: I −24 HbA1c <6.5%/48 mmol/mol without diabetic medications: I 79% PP, 75% ITT Difference from baseline and 6 months. HbA1c <6.5%/48 mmol/mol without diabetic medications: I 35% PP, 30% ITT Difference from baseline and 1 year.HbA1c <6.5%/48 mmol/mol without diabetic medications: I 24% PP, 20% ITT
Taheri et al.^36^ Qatar DIADEM‐1 study	147	1 year	Intensive lifestyle intervention: 49/21, 55/70, 7.0/52.5, 100.6 kg, 21.9 months Usual care: 58/19, 67/77, 7.0/52.5, 101.7 kg, 20.5 months	Type 2 diabetes, age 18–50, short duration diabetes (≤3 years), BMI ≥ 27.	Intensive lifestyle intervention:Total diet replacement (12 weeks—800–820 kcal/day) and 12‐week food reintroduction. Dietitian and personal trainer review every 2 weeks in this phase. Physical activity—aim for 10,000 steps/day and then 150 min/week. Weight loss maintenance phase 6 months of self‐management. Diabetes medications discontinued at start of intervention and re‐introduced based on guidelines. Antihypertensive and lipid medications monitored. Control: Usual care	Difference from baseline and year 1. Mean weight change (kg): I −12.0, C −4.0 (*p* < 0.0001) Mean HbA1c% change: I −0.9, C −0.4 (*p* = 0.020) Mean HbA1c mmol/mol change: I −9.50, C −3.46 (*p* = 0.020) HbA1c <6.5%/48 mmol/mol without diabetic medications: I 61%, C 12% *p* < 0.0001)

Abbreviations: BMI, body mass index in kg/m^2^; C, control; DSE, diabetes support and education; I, intervention; ILI, intensive lifestyle intervention; ITT, intention to treat; LCD, low‐calorie diet; PP, per protocol; VLCD, very‐low‐calorie diet.

### 3.1 Diabetes remission rates

Lean et al.^27^ conducted a clustered randomized controlled trial to assess if intensive management of weight through 3–5 months of total diet replacement (825–853 kcal/day) and stepped food re‐introduction could lead to remission of diabetes in a primary care setting (the DiRECT trial). Initial results were presented at 1 year^27^ and achieved a weight loss of 10 kg in the intervention group compared with 1.0 kg in the control group (*p* < 0.001). In the second year of the study,^28^ weight gain was treated with titrated meal replacements depending on if the gain was more than 2 or 4 kg. At this point, the weight loss was 7.6 kg in the intervention group compared with 2.3 kg in the control group (*p* < 0.001). Strengths of the study include the number of participants included (*n* = 298), the duration of follow‐up (24 months), and the real‐world setting, so the results could be translated to community populations with T2D. Limitations of the study include the lack of blinding of participants and assessors (although difficult to achieve with any dietary intervention and this risk of bias applies to all included studies). Participants receiving insulin were excluded, and this is an important subpopulation, for whom it is important to quantitate the possibility of remission. The definition of remission was HbA1c <6.5% off medications for at least 2 months which is a shorter duration than the standard of 1 year used in many studies which potentially produced a higher remission rate. The study population was of homogenous ethnicity with 98% being white in the intervention arm and 99% in the control arm. The authors commented that the results may not be able to be extrapolated to a South Asian population. They noted that as de‐prescribing of diabetic medication is not part of standard guidelines, it is possible that a higher proportion of the control group may have achieved remission if this was done. The authors found a remission rate of diabetes of 46% in the intervention group compared with 4% in the control group at 1 year (*p* < 0.001). At 2 years, remission rates in the intervention group were 35.6% and 3.4% in the control group (*p* < 0.0001).

The DIADEM‐1 study, presented by Taheri et al.^36^ was a randomized controlled trial comparing an intensive lifestyle intervention to standard care. The cohort was younger than the DiRECT study (age 18–50), had a shorter duration of diabetes (less than 3 years), and there was a male preponderance (73%). Total dietary replacement of 800–820 kcal/day for 12 weeks followed by a phased food re‐introduction and increased physical activity resulted in weight loss of 12.0 kg in the intervention group compared with 4.0 kg in the control group (*p* < 0.0001). Strengths of the study include the number of participants (*n* = 147), the duration of the study (12 months), and the inclusion of patients taking insulin who had been excluded from the DiRECT trial. A limitation noted by the author was that the intervention was delivered by a multidisciplinary team that may not be available in other health settings. The remission rate was 61% in the intervention group at 12 months compared to 12% in the control group (odds ratio 12.03 [95% CI 5.17–28.03], *p* < 0.0001).

Reid‐Larsen et al.^32^ performed a secondary analysis of a randomized controlled trial^33^ examining the effect of energy restriction for 4 months (amount unspecified) and supervised exercise for glycemic control compared with standard care. In this analysis, the primary outcome was partial or complete remission of T2D. Weight loss at 2 years was 1.4 kg in the intervention group and 0.8 kg in the control group (*p* = 0.17). Strengths of this study include the number of participants (*n* = 98) and length of follow‐up. The trial was well conducted and the participants represented the source population. Weaknesses include the energy restriction only occurring for the first 4 months of the study and the location of much of the delivery being a teaching hospital which may not be representative of the care that the average patient with T2D is able to access. Also, the secondary analysis was not powered to assess T2D remission rates. Remission rates in this study were larger at 23% for the intervention group than 7% for the control group, but the difference was not statistically significant (*p* = 0.08).

A retrospective analysis was conducted using the results of the Look AHEAD trial.^30^, ^31^ The trial lasted 4 years. Energy restriction ranged from 1200 to 1800 kcal/day in the intervention group and meal replacements were offered. Physical activity was increased to 175 min/week. Weight loss at year 1 was 8.6% in the intervention group compared to 0.7% in the control group (*p* < 0.001). In year 4, weight loss was 4.7% in the intervention group compared with 0.8% in the control group (<0.001). Strengths of the study again include the number of participants (*n* = 5145) and the duration of the study. This was also an innovative and pivotal study in reframing T2D from a chronic progressive condition to one with a possibility of remission. The analysis excluded participants who underwent bariatric surgery. However, they also excluded participants with missing outcome data allowing for potential attrition bias. The patients were extensively screened for their potential to lose weight, meaning the results may not be applicable to the general population with T2D. Also, as it was a retrospective analysis, a power calculation was not done. At year 1, 11.5% in the intervention group compared to 2% in the control group achieved remission (*p* < 0.001). At year 4, this was 7.3% in the intervention group and 2% in the control group (*p* < 0.001).

A single‐arm study to assess the efficacy, safety, and durability of a very‐low‐calorie diet (VLCD) in Thai patients for the remission of T2D was undertaken by Umphonsathien and colleagues.^35^ The authors recruited members of staff at a hospital. The intervention involved a run‐in of 2 weeks of total diet replacement of 600 kcal/day to assess compliance. If this was tolerated, it was followed by 8 weeks of 600 kcal/day, then a stepwise calorie increase with week 9—800 kcal/day, week 10—1000 kcal/day, week 11 —1200 kcal/day, and week 12—1500 kcal/day. Mean weight loss of 9.5 kg was achieved in the study group at 14 weeks. Strengths include the clear outcomes and aims of the study and that a power calculation was done. Weaknesses include the small sample size (*n* = 20), no control group, the recruitment of hospital staff who may not be representative of the typical population with T2D and participants’ level of compliance was unclear. Rates of remission were 75% at 14 weeks, and fell to 30% and 6 months and 20% at 1 year.

A 6‐month single‐arm study was undertaken in participants with newly diagnosed diabetes to assess the effect of a program including a 500 kcal/day deficit and exercise (walking) on the rate of remission.^34^ Mean weight loss at 6 months was 9.7 kg in the participants. Strengths of this study by Ades et al. include the follow‐up time (6 months) and the clear aims and methods reported. Weaknesses include the fact that there was no control arm, it is unclear how participants were recruited and there were a small number of participants (*n* = 12). The rate of remission was very high at 80%. There is, however, no longer term follow‐up to assess how participants fared once the intervention was complete.

Sarathi et al.^29^ conducted a single‐arm study on young adults with newly diagnosed T2D to assess the reversibility of the condition. Calorie intake was limited to 1500 kcal/day and participants undertook 1 h of brisk walking daily. Mean weight loss was 6.6 kg at 1 year and 7.7 kg at 2 years. Strengths include clear aims and interventions of the study. Weaknesses include the small sample size (*n* = 32), a lack of control arm, no power calculation, and the recruitment process was unclear. Therefore, the adults recruited may not be typical of the population with T2D. The remission rate at 1 year was 75% and at 2 years 68.8%. This is an impressive maintenance rate of remission. The calorie restriction (1500 kcal) was not as low as the DiRECT study or Umphonsathien et al.^35^

Bhatt et al.^24^ conducted a single‐arm study on the use of 1000 kcal/day total diet replacements for 12 weeks. Moderate aerobic and resistance intensity exercise was advised from week 2. Mean weight loss at 12 weeks was 6.8 kg. This was a poorly conducted and reported study. There were no clearly described aims and outcomes. There was no control arm and the sample size was small (*n* = 12). It was unclear how participants were recruited. Fifty percent of participants achieved remission of diabetes at 12 weeks.

### 3.2 Intensity of weight loss intervention

Three factors varied in the intensity of the intervention: the level of calorie restriction, the duration of calorie restriction, and the addition of supervised structured physical activity. A comparison of the intensity of the intervention is tabulated in Table S4. An average adult's calorie intake is assumed to be 2250 kcal/day (males 2500 kcal and females 2000 kcal),^37^ the calorie deficit has been calculated as the difference between average calorie intake and the calorie intake of the intervention. This is not accounting for cultural differences in calorie intake or the number of males and females in each study.

One of the most intense calorie restrictions was in the DiRECT study,^27^, ^28^ but there was also a maintenance plan beyond the first intervention. Of note, weight losses had decreased on average by year 2 from −10.0 to −7.6 kg. The DIADEM‐1 study^36^ combined a strict calorie restriction with physical exercise and achieved the highest total weight loss at the end of a study. Umphonsathien et al.^35^ had the most severe calorie restriction but there was no intervention beyond 14 weeks and weight loss results were not collected beyond this. There were relatively low calorie restrictions by Ades et al.^34^ and Sarathi et al.^29^ but impressive weight losses. There were ongoing weight losses in the second year for Sarathi et al.^29^ Both these studies used physical activity raising the possibility of its importance in remission maintenance. However, contradictory to this hypothesis is that Ried‐Larsen et al.^32^ used structured exercise as part of the intervention with minimal weight loss and a non‐statistically significant reduction in remission in the control group. The calorie restriction only lasted 4 months in this study, and it is possible that this was not a strong enough intervention. The optimum balance of caloric restriction and exercise for diabetes remission and maintenance is an important question that needs further assessment. Weight loss was expressed as a percentage by Gregg et al.^30^ so it is difficult to directly compare with the other studies.

### 3.3 Remission rates compared with weight loss

Figure 1 shows weight loss in kilograms plotted against remission rates for the intervention groups as well as the control groups. Gregg et al.^30^ was excluded from this as weight loss was presented in percentage terms. Increased weight loss appears to be associated with increased rates of remission (*r* = 0.83). The largest mean weight losses were made by Lean et al.^27^ in year 1 at 10 kg, Taheri et al.^36^ in year 1 at 12.0 kg, Ades et al.^34^ at 9.7 kg at just 6 months, and Umphonsathien et al.^35^ at just 14 weeks with 9.5 kg. The remission rate fell rapidly over the first year for Umphonsathien et al.^35^ and by just over 10% for Lean et al.^28^ Lean et al.^27^ found that the odds ratio per kilogram weight loss for attaining remission was 1.32 (95% CI: 1.23– .41; *P* < 0.0001). Gregg et al.^30^ found that the probability of 1‐year remission was the highest among those who lost greater than 6.5% weight (16.4%; 95% CI: 14.5%–18.6%). Remission of diabetes was possible for 70% of those who maintained more than 15 kg weight loss in the DiRECT study^27^; however, only 24% was able to achieve this weight loss in the first year despite a severe calorie restriction. Sarathi et al.^29^ found that no patients who achieved remission and maintained or decreased from their 3‐month weight loss had recurrence of T2D. Gregg et al.^30^ found that one‐third of those who had a remission returned to diabetes every year. The associated weight gain was not reported.

### 3.4 Change in HbA1c levels

This data is presented in Table S5. The studies with higher baseline HbA1c level had a greater fall (Sarathi et al.^29^ and Bhatt et al.^24^). Gregg et al.^30^ did not present data on changes in HbA1c levels. Interestingly, Ried‐Larsen et al.^32^ found an increase in HbA1c level in both the intervention and control groups at 24 months. The 12‐month data from this study,^33^ showed a change of −0.31% in the intervention group and −0.08% in the control group (*p* = 0.15). As some patients developed remission, presumably in those that did not, there was an overall increase in HbA1c to account for this mean rise in each arm.

There is no clear association between baseline HbA1c levels and remission rate (*r* = 0.36) (Figure S2). Gregg et al.^30^ found an association between low baseline HbA1c levels and remission rates (*p* < 0.001). Umphonsathien et al.^35^ and Sarathi et al.^29^ found no association between baseline HbA1c and remission rates. Of note, Ried‐Larsen et al.^32^ and Taheri et al.^36^ had particularly low levels of baseline HbA1c in the intervention and control groups. This may explain the relatively low levels of mean weight loss achieving better remission rates in these control groups (7% and 12%) compared with the control groups in the other studies. This may also have contributed to the lack of statistical significance between the intervention and control groups for Ried‐Larsen et al.^32^

### 3.5 Remission compared to and baseline weight and duration of diabetes

Figures S3 and 2 have been plotted to show rates of remission in the intervention arm against baseline weight and duration of diabetes Only the first remission rate recorded (which was the highest rate in all studies) has been plotted to examine the relationship between the maximal remission potential and the other variables. There is no clear association between baseline weight and remission rates (*r* = −0.43). Ethnically, populations such as Thai may have lower mean body weights. Lean et al.^28^ found no association between baseline BMI and likelihood of remission at 24 months. Umphonsathien et al.^35^ and Sarathi et al.^29^ found no association between baseline weight and remission rates. However, Gregg et al.^30^ did find an association with lower BMI and likelihood of further remission (*p* < 0.001).

**FIGURE 2:**
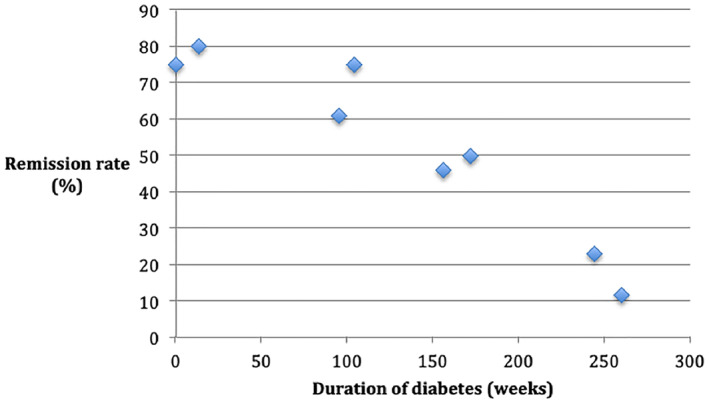
Remission rate compared with duration of diabetes

Figure 2 shows an inverse association between the duration of diabetes and the remission rate (*r* = −0.94). The duration of diabetes was a mean value for all studies, except Umphonsathien et al.^35^ Gregg et al.^30^ and Bhatt et al.^24^ who presented median values. The studies with the longest duration since diagnosis were Gregg et al.^30^ and Ried‐Larsen et al.^32^ This may have contributed to the lower remission rates in both of these studies. There was no association between the duration of diabetes within 6 years from diagnosis inclusion criteria in the DiRECT study. However, Figure 2 shows the highest rates of remission were achieved in those newly diagnosed and the mean time since diagnosis was 3 years for both arms of the DiRECT study. Both studies with participants having newly diagnosed diabetes (Sarathi et al.^29^; Ades et al.^34^) were single‐arm studies with evidence of bias (Table S2). Gregg et al.^30^ found a statistically significant association between fewer years since diagnosis and remission rates (*p* < 0.001). Bhatt et al.^24^ found that responders had a shorter duration of diabetes than non‐responders (1.5 vs. 4.0 years), but no statistical analysis was carried out. In contrast, Umphonsathien et al.^35^ found no association between diabetes duration and remission rates.

### 3.6 Adverse events

Taheri et al.^36^ found fewer serious adverse events in the intervention arm compared to the control arm as did Lean et al.^28^ who also reported statistical significance with *p* = 0.029 (see Table 3). There were no significant differences in adverse events between the two groups reported by Gregg et al.^30^ Bhatt et al.^24^ Sarathi et al.^29^ and Ades et al.^34^ did not describe adverse events. Umphonsathien et al.^35^ had no adverse events and Reid‐Larsen et al.^32^ had one serious adverse event in the intervention group (an episode of atrial fibrillation), but this was not statistically significant (*p* = 1.00). There were few adverse events described that related to the interventions and none that led to withdrawal from the studies.

**TABLE 3:** Serious adverse events

Author, Year	Serious adverse events		
	Lifestyle/Reduced calorie diet arm (*n*)	Control (*n*)	*p*‐Value
Lean et al.^27^	*n* = 7 Two events in the same patient thought to be related to intervention—biliary colic and abdominal pain. Others were brief overnight admissions for unrelated investigations/events. None led to withdrawal from the trial.	*n* = 2 Including categories of infection and infestation, wound infection, nervous system disorders, seventh nerve paralysis.	Non‐significant (described in Lean et al., 2019)
Lean et al.^28^Year 2 results	*n* = 9 events in 6 participants One non‐fatal MI in a patient that did not attend for review. None led to withdrawal from the trial.	*n* = 22 events in 16 participants x2 cerbrovascular accident, toe amputation, aortic aneurysm rupture, sudden death.	0.029
Bhatt et al.^24^	Not described.		
Sarathi et al.^29^	Not described.		
Gregg et al.^30^	Not described for subpopulation examined in this analysis.		
Ried‐Larsen et al.^32^ Johansen et al.^33^	*n* = 1 Atrial fibrillation.	*n* = 0	1.00
Ades et al.^34^	Not described		
Umphonsathien et al.^35^	*n* = 0		
Taheri et al.^36^	*n* = 0	*n* = 55 events in 4 participants 4 admissions to hospitals for unexpected event—supraventricular tachycardia, abdominal pain, pneumonia and epididymo‐orchitis. 1 expected event—hyperglycemia	Not reported

## 4 DISCUSSION

This review has found that dietary energy restriction is an effective treatment strategy for the remission of T2D with higher rates associated with increased weight loss (*r* = 0.83), decreased time from diagnosis (*r* = −0.94), and increased intensity of intervention (in terms of level and duration of calorie restriction) in studies where diagnosis was not recent. However, the durability of extreme calorie restriction beyond 2 years still needs to be established. The highest remission rates involved patients with a new diagnosis of T2D although these studies had a high level of bias. Five studies presented remission data at 1 year and the remission rate varied between 11.5% and 75%.^27^, ^29^, ^30^, ^35^, ^36^ Energy restriction as a method of diabetes reversal has also been found to be safe with relatively few serious adverse events reported.

Bariatric surgery has been recommended as a treatment for people with T2D and a BMI of 30 kg/m^2^ or above, or at a lower level if of Asian origin and not achieving treatment targets.^38^ Much of the published research has compared surgery to intensive weight management. Parikh et al.^39^ found superior rates of remission with surgery (65%) compared with medical weight management (0%) at 6 months (*p* < 0.0001). Medical weight management included tailored counselling on diet, exercise and adjustment of diabetic medications. Cummings et al.^40^ compared Roux‐en‐Y gastric bypass with an intensive lifestyle and medical intervention and found 60% remission rates in the surgery arm and 5.9% in the medical arm at 1 year. The intensive lifestyle arm in this study included dietary advice, exercise five times a week, and diabetic pharmaceutical treatment in keeping with current guidelines. Courcoulas et al.^41^ found that Roux‐en‐Y gastric bypass was superior (50%) to a laparoscopic gastric band (27.3%) and an intensive lifestyle weight loss intervention (0%) at 1 year. Courcoulas et al.^41^ reported that the medical intervention was based on the Look AHEAD trial, but the remission rate was much lower than that reported by Gregg et al.^30^ Parikh et al.^39^ mentioned themselves that they could have integrated more extensive lifestyle changes into the medical weight management arm.

A 7‐year multicenter longitudinal study^42^ found that the rate of remission was 60.2% for Roux‐en‐Y gastric bypass and 20.3% for laparoscopic gastric banding in patients with T2D. The authors reported that lifestyle programs are often the first‐line recommendations for obesity management, but are largely ineffective and when successful, weight loss is rarely sustained. However, the outcomes in the lifestyle arms of bariatric surgery are much below those of studies primarily focused on remission of diabetes through caloric restriction in this review. This suggests the intensity of caloric‐restriction intervention may be too low in these bariatric trials. The outcomes in many of the calorie‐restricted studies in this review were comparable to the remission rates achieved by bariatric surgery.

Carbohydrate restriction has also been used as a treatment for T2D, but meta‐analysis has shown little effect on glycemic control beyond the short term.^43^ However, a more recent study^44^ compared a low‐carbohydrate diet (moderate protein, fat to satiety, and no energy restriction), that aimed for ketosis with usual care in T2D and 60% of completers achieved an HbA1c below 6.5% without diabetic medication (excluding metformin) at 1 year. At 2 years, 54% of completers had an HbA1c below 6.5% with no diabetic medication except metformin.^45^ Therefore, it is clear that further routes of diabetes remission beyond bariatric surgery are emerging.

National guidance^9^ briefly mentions lifestyle changes as advice that should be offered to patients, with the focus of the guideline being on an algorithm of increasing medication. All the studies examined in this review have found low levels of remission with standard care.

Dambha‐Miller et al.^46^ conducted a 5‐year prospective cohort study including 867 participants with newly diagnosed T2D. Remission was achieved in 30% at 5‐year follow‐up of the pooled data and the risk ratio was significantly higher in those that achieved ≥10% weight loss (in years 1–5), than those that maintained the same weight (risk ratio 2.43 [95% CI: 1.78–3.31; *p* < 0.01]). The remissions in this study were achieved without extreme calorie restriction or lifestyle interventions. Ten percent weight loss seems a reasonable initial target for remission of diabetes and was the minimum weight loss that resulted large levels of remission in a number of studies.^27^, ^28^, ^29^, ^34^, ^35^, ^36^ In fact, in the DiRECT study with a baseline weight of approximately 100 kg in both groups, of those losing greater than 10 kg, 64% achieved remission at 24 months. For 5–10 kg, it was 29%, and for less than 5 kg, it was 5% remission. Remission was more likely with greater weight loss from baseline with adjusted odds ratio 1.2 per kg loss. The most impressive remissions in the current review were achieved by Sarathi et al.^29^ and Ades et al.^34^ who both conducted studies on patients with newly diagnosed diabetes. The calorie restrictions in both studies were relatively low, but perhaps more easily maintained.

Kelly and colleagues^17^ found that when they classified lifestyle interventions into therapeutic (600–1500 kcal total daily intake), the weighted mean remission was 49.4% versus subtherapeutic (800–1500 kcal total daily intake), where the weighted mean remission was 6.9%. The distinction between therapeutic and subtherapeutic dosing was based on treatment goals and whether the intervention produced remission in a substantial proportion of participants. There is clearly an overlap in calorie intake in both groups. They did not examine the relationship between time from diagnosis of T2D with remission rates. The authors noted that the dosing intensity required to achieve remission of T2D was much higher than that required to prevent it. It is therefore plausible that a lower intensity intervention may be adequate to achieve remission earlier in the pathogenesis of the disease (i.e., at diagnosis) where there is less *β* cell damage. In an era of COVID‐19 infection where the risk of diabetes is even greater than previously thought, would the information that a new diagnosis of diabetes is likely reversible for a limited number of years be the motivating factor to help patients lose and maintain weight loss? This is an interesting area for future research.

Lean et al.^27^ noted that medication withdrawal is not part of standard guidelines which may have led to the rate of remission being under‐reported in their control group. If 30% of patients with newly diagnosed diabetes are achieving remission without intensive interventions,^46^ and many first and second intensification medications lead to weight gain, perhaps national guidelines should include instructions for formal de‐prescribing, to aid those who are managing to lose weight to maintain remission.

Strengths of this review include the fact that there have been few reviews in this developing area of research. There were limitations of time and resources and the review would have benefited from wider searches of grey literature and unpublished research. Also, the results would have benefited from the calculation of meta‐analysis of odds ratios to quantify associations and calculate the statistical significance if more resources were available. A paucity of well‐constructed randomized controlled trials in this developing area of research meant that a small number of single‐arm studies were included which may increase the risk of bias in the results and conclusions.

There was great heterogeneity in the definition of remission in the studies but a pragmatic definition of any duration of HbA1c <6.5%/48 mmol/mol without medication was used for the purposes of this review. This would have led to bias as studies that only measured remission at 1 year may be underreporting maximal remission rates at any time point.

## 5 CONCLUSION

Recommendations for practice based on the findings of this review are to offer patients with a diagnosis of diabetes within the last 6 years an opportunity to achieve remission. The target weight loss should be at least 10% of body weight in individuals with sufficient overweight and obesity. The high levels of remission of newly diagnosed diabetes were a surprising finding, as this was not reported in the highly publicized DiRECT trial. Therefore, weight loss should be particularly encouraged as a first‐line approach for those with a new diagnosis of T2D. The favored method should reflect patient preferences, but options should include an energy‐restricted diet. Total meal replacement methods have been very effective in a number of studies in achieving this in the short and medium terms, but are not the exclusive route to remission. More research is needed to establish the optimal intensity in terms of energy restriction and duration, and the role of exercise for the maintenance of remission. National guidelines should be updated to reflect recent evidence.

## CONFLICT OF INTEREST

The authors have no conflict of interest.

## AUTHORS' CONTRIBUTION

Elizabeth Jacob was involved in the initial data collection, analysis and interpretation. Amanda Avery was involved in critical revision of the article. Both authors contributed substantially to the intellectual content during manuscript draft or revision. All authors approved the manuscript and the submission.

## Supporting information

Supplementary Information
