# Supplementary material for: Energy‐restricted interventions are effective for the remission of newly diagnosed type 2 diabetes: A systematic review of the evidence base
Source: Obes Sci Pract. 2021 May 15;7(5):606–18. doi: 10.1002/osp4.504 (PMC8488441; doi:10.1002/osp4.504)
Supplement: Supplementary file 1 — Supplementary Information [file OSP4-7-606-s001.docx]

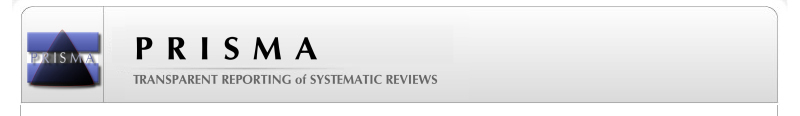
**PRISMA 2009 Flow Diagram**

Studies included in analysis
(n = 8)

Records excluded
(n = 624)

Records screened
(n = 641 )

Records after duplicates removed
(n = 641)

Additional records identified through other sources
(n = 1)

## Identification

## Eligibility

## Included

## Screening

Records identified through database searching
(n = 660)

Full-text articles excluded, with reasons
(n = 9)

Drugs intervention in calorie-reduced arm x2

Studies on children x1

Cohort study x1

No outcome of HbA1c <6.5% off diabetes medication x1

Prevention/prediabetes x2

Calorie restriction with surgery x1

No clear energy restriction x1

**Figure S1: Flowchart - adapted from the prisma flow diagram by Moher et al.^25^**

Studies assessed for eligibility
(n = 17)
